# Correction: Nafion film transport properties in a low-Pt PEM fuel cell: impedance spectroscopy study

**DOI:** 10.1039/d1ra90053f

**Published:** 2021-02-08

**Authors:** Tatyana Reshetenko, Andrei Kulikovsky

**Affiliations:** Hawaii Natural Energy Institute, University of Hawaii Honolulu Hawaii 96822 USA tatyanar@hawaii.edu; Theory and Computation of Energy Materials (IEK-13), Forschungszentrum Jülich GmbH, Institute of Energy and Climate Research D-52425 Jülich Germany A.Kulikovsky@fz-juelich.de

## Abstract

Correction for ‘Nafion film transport properties in a low-Pt PEM fuel cell: impedance spectroscopy study’ by Tatyana Reshetenko *et al.*, *RSC Adv.*, 2019, **9**, 38797–38806, DOI: 10.1039/C9RA07794D.

The authors would like to propose a modified version of the model presented in their article, which is consistent with the idea of the 1d + 1d approach reported in ref. 1. This adjustment makes the model more sensitive to the Nafion film parameters.

In this Correction article, equation numbers and notations referred to in the text are those used in the original article. On the right side of eqn (3) and its dimensionless version, eqn (12), the oxygen flux is calculated at the metal surface. A more accurate approximation is to use the flux *N*_N,p_ at the open pore radius *R*_p_ instead of *N*_N,m_, *i.e.*,1
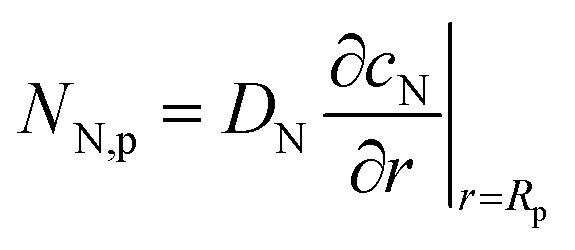
and its dimensionless variant should be substituted into eqn (3) and (12), respectively. It follows that in eqn (23) the perturbed flux 
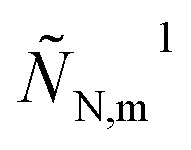
 should be replaced by 
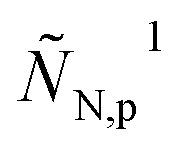
 and eqn (23) takes the form2
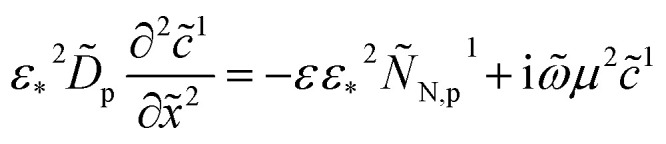


The flux 
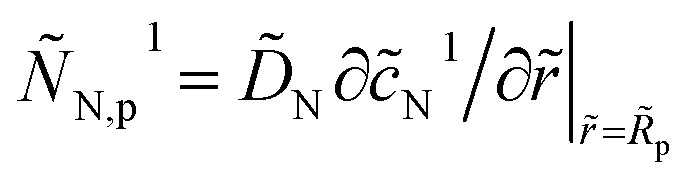
 is obtained from solving eqn (24), resulting in3
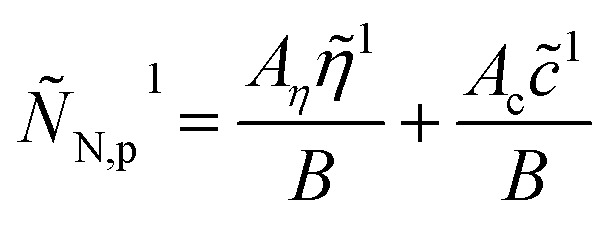
where4
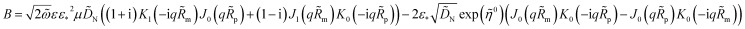
5
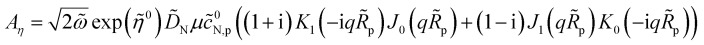
6
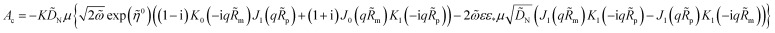
and7
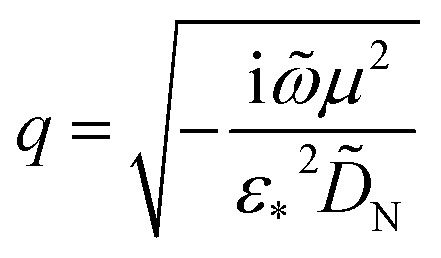
Here, *J*_0_, *J*_1_ are the Bessel functions of the first kind, and *K*_0_, *K*_1_ are the modified Bessel functions of the second kind. This modification leads to marginal changes in the calculation results reported in the original article. In the range of current densities 100 to 800 mA cm^−2^, the Nafion film thickness changes between 8.5 and 12 nm and the film resistivity decreases from 0.6 s cm^−1^ down to 0.25 s cm^−1^, in agreement with the results in the original article.

Qualitatively, the pore radius is about an order of magnitude larger than the Nafion film thickness, meaning that the shape of the small film element is close to planar and hence the real part of the fluxes 
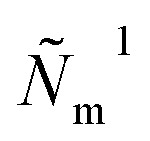
 and 
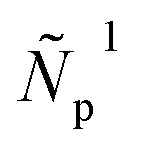
 are almost equal. Eqn (23) takes into account the phase shift due to transport of 
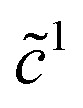
 perturbation through the film. However, replacing *N*_N,m_ by *N*_N,p_ in this equation increases the effect of the film on the phase shift. In general, this makes the model more sensitive to the film parameters.

The Royal Society of Chemistry apologises for these errors and any consequent inconvenience to authors and readers.

## Supplementary Material
